# Supplementary material for: Modeling glioblastoma heterogeneity as a dynamic network of cell states
Source: Mol Syst Biol. 2021 Sep 16;17(9):e10105. doi: 10.15252/msb.202010105 (PMC8444284; doi:10.15252/msb.202010105)
Supplement: Supplementary file 6 — Source Data for Figure 5 [file MSB-17-e10105-s004.zip › Figure5A_sourcedata/GSEA_3071/nosigdb_stateIV.GseaPreranked.1622795164626/SINGH_SOX2_UPREGULATED_TRANSCRIPTS_PMID_28122245.html]

Details for gene set SINGH\_SOX2\_UPREGULATED\_TRANSCRIPTS\_PMID\_28122245[GSEA]

|  || Dataset | state53071 |
| Phenotype | NoPhenotypeAvailable |
| Upregulated in class | na\_pos |
| GeneSet | SINGH\_SOX2\_UPREGULATED\_TRANSCRIPTS\_PMID\_28122245 |
| Enrichment Score (ES) | 0.4454884 |
| Normalized Enrichment Score (NES) | 2.8653872 |
| Nominal p-value | 0.0 |
| FDR q-value | 0.0 |
| FWER p-Value | 0.0 |
Table: GSEA Results Summary

  

Fig 1: Enrichment plot: SINGH\_SOX2\_UPREGULATED\_TRANSCRIPTS\_PMID\_28122245      
 Profile of the Running ES Score & Positions of GeneSet Members on the Rank Ordered List

  

| PROBE | GENE SYMBOL | GENE\_TITLE | RANK IN GENE LIST | RANK METRIC SCORE | RUNNING ES | CORE ENRICHMENT || 1 | LMO1 |  |  | 2 | 2.803 | 0.0135 | Yes |
| 2 | C1orf61 |  |  | 3 | 2.760 | 0.0272 | Yes |
| 3 | ID4 |  |  | 7 | 2.594 | 0.0395 | Yes |
| 4 | PTPRZ1 |  |  | 10 | 2.539 | 0.0517 | Yes |
| 5 | NFIA |  |  | 24 | 2.181 | 0.0600 | Yes |
| 6 | PTN |  |  | 25 | 2.140 | 0.0706 | Yes |
| 7 | FAM181B |  |  | 26 | 2.105 | 0.0810 | Yes |
| 8 | SCRG1 |  |  | 28 | 2.056 | 0.0910 | Yes |
| 9 | LINC00461 |  |  | 30 | 2.010 | 0.1008 | Yes |
| 10 | MAP3K1 |  |  | 42 | 1.903 | 0.1081 | Yes |
| 11 | KAT6B |  |  | 54 | 1.813 | 0.1149 | Yes |
| 12 | MAP2 |  |  | 55 | 1.811 | 0.1239 | Yes |
| 13 | SOX11 |  |  | 56 | 1.811 | 0.1329 | Yes |
| 14 | CTH |  |  | 60 | 1.774 | 0.1411 | Yes |
| 15 | SYT11 |  |  | 67 | 1.740 | 0.1485 | Yes |
| 16 | CDH2 |  |  | 68 | 1.735 | 0.1571 | Yes |
| 17 | TUBB2B |  |  | 69 | 1.731 | 0.1657 | Yes |
| 18 | POU3F2 |  |  | 70 | 1.728 | 0.1743 | Yes |
| 19 | KLHDC8A |  |  | 73 | 1.710 | 0.1824 | Yes |
| 20 | DCLK2 |  |  | 74 | 1.704 | 0.1909 | Yes |
| 21 | DPYSL3 |  |  | 75 | 1.691 | 0.1993 | Yes |
| 22 | ZBTB20 |  |  | 81 | 1.628 | 0.2063 | Yes |
| 23 | TCF4 |  |  | 96 | 1.551 | 0.2113 | Yes |
| 24 | GPM6A |  |  | 98 | 1.538 | 0.2187 | Yes |
| 25 | ROBO2 |  |  | 114 | 1.499 | 0.2232 | Yes |
| 26 | NFIB |  |  | 117 | 1.491 | 0.2302 | Yes |
| 27 | TTC28 |  |  | 119 | 1.490 | 0.2374 | Yes |
| 28 | TRIM9 |  |  | 125 | 1.470 | 0.2437 | Yes |
| 29 | NCAM1 |  |  | 129 | 1.456 | 0.2503 | Yes |
| 30 | LSAMP |  |  | 130 | 1.454 | 0.2575 | Yes |
| 31 | RGS12 |  |  | 133 | 1.439 | 0.2643 | Yes |
| 32 | TUBA1A |  |  | 165 | 1.337 | 0.2647 | Yes |
| 33 | TNIK |  |  | 172 | 1.312 | 0.2701 | Yes |
| 34 | ETV5 |  |  | 174 | 1.305 | 0.2763 | Yes |
| 35 | TCF12 |  |  | 176 | 1.303 | 0.2826 | Yes |
| 36 | NKAIN3 |  |  | 183 | 1.295 | 0.2878 | Yes |
| 37 | CDCA7L |  |  | 185 | 1.292 | 0.2941 | Yes |
| 38 | LCORL |  |  | 186 | 1.289 | 0.3005 | Yes |
| 39 | SOX6 |  |  | 207 | 1.244 | 0.3027 | Yes |
| 40 | GOLM1 |  |  | 209 | 1.240 | 0.3086 | Yes |
| 41 | SOX2 |  |  | 212 | 1.231 | 0.3143 | Yes |
| 42 | SOX9 |  |  | 214 | 1.223 | 0.3202 | Yes |
| 43 | SHC3 |  |  | 215 | 1.218 | 0.3263 | Yes |
| 44 | LDLRAD3 |  |  | 216 | 1.218 | 0.3323 | Yes |
| 45 | NOVA1 |  |  | 234 | 1.181 | 0.3348 | Yes |
| 46 | KIF21A |  |  | 239 | 1.171 | 0.3398 | Yes |
| 47 | WSCD1 |  |  | 253 | 1.141 | 0.3429 | Yes |
| 48 | GAB1 |  |  | 257 | 1.133 | 0.3479 | Yes |
| 49 | SALL1 |  |  | 262 | 1.122 | 0.3527 | Yes |
| 50 | NLGN1 |  |  | 282 | 1.069 | 0.3542 | Yes |
| 51 | FOXO3 |  |  | 291 | 1.054 | 0.3579 | Yes |
| 52 | SRI |  |  | 298 | 1.037 | 0.3618 | Yes |
| 53 | ITGB8 |  |  | 307 | 1.027 | 0.3653 | Yes |
| 54 | PBX1 |  |  | 332 | 0.987 | 0.3655 | Yes |
| 55 | LYPD1 |  |  | 338 | 0.980 | 0.3693 | Yes |
| 56 | MSI2 |  |  | 354 | 0.971 | 0.3712 | Yes |
| 57 | SPECC1 |  |  | 360 | 0.960 | 0.3749 | Yes |
| 58 | FOXG1 |  |  | 363 | 0.950 | 0.3793 | Yes |
| 59 | MLC1 |  |  | 366 | 0.946 | 0.3836 | Yes |
| 60 | CDKN1B |  |  | 374 | 0.939 | 0.3868 | Yes |
| 61 | SOX4 |  |  | 377 | 0.928 | 0.3910 | Yes |
| 62 | ZEB1 |  |  | 385 | 0.920 | 0.3942 | Yes |
| 63 | OLIG1 |  |  | 391 | 0.914 | 0.3978 | Yes |
| 64 | CELSR2 |  |  | 428 | 0.875 | 0.3950 | Yes |
| 65 | OSBPL6 |  |  | 430 | 0.871 | 0.3991 | Yes |
| 66 | RFTN2 |  |  | 432 | 0.868 | 0.4032 | Yes |
| 67 | FAT3 |  |  | 440 | 0.864 | 0.4061 | Yes |
| 68 | TRIL |  |  | 445 | 0.859 | 0.4096 | Yes |
| 69 | SMAD1 |  |  | 448 | 0.857 | 0.4134 | Yes |
| 70 | ZNF608 |  |  | 478 | 0.832 | 0.4118 | Yes |
| 71 | DCAF16 |  |  | 482 | 0.829 | 0.4153 | Yes |
| 72 | ETV1 |  |  | 488 | 0.824 | 0.4184 | Yes |
| 73 | SOX21 |  |  | 489 | 0.823 | 0.4225 | Yes |
| 74 | APC |  |  | 492 | 0.822 | 0.4262 | Yes |
| 75 | DOK5 |  |  | 505 | 0.814 | 0.4278 | Yes |
| 76 | S100A16 |  |  | 521 | 0.797 | 0.4288 | Yes |
| 77 | BCL7A |  |  | 548 | 0.765 | 0.4274 | Yes |
| 78 | SCD5 |  |  | 555 | 0.759 | 0.4300 | Yes |
| 79 | SPATA6 |  |  | 559 | 0.756 | 0.4332 | Yes |
| 80 | WDR82 |  |  | 564 | 0.751 | 0.4361 | Yes |
| 81 | PTOV1 |  |  | 580 | 0.740 | 0.4368 | Yes |
| 82 | VPS37B |  |  | 584 | 0.739 | 0.4399 | Yes |
| 83 | CDH4 |  |  | 610 | 0.721 | 0.4385 | Yes |
| 84 | GLCCI1 |  |  | 611 | 0.719 | 0.4421 | Yes |
| 85 | STIM2 |  |  | 639 | 0.704 | 0.4402 | Yes |
| 86 | JAKMIP2 |  |  | 657 | 0.685 | 0.4402 | Yes |
| 87 | SETD5 |  |  | 681 | 0.672 | 0.4390 | Yes |
| 88 | ATF7IP |  |  | 711 | 0.653 | 0.4365 | Yes |
| 89 | POU2F1 |  |  | 718 | 0.649 | 0.4385 | Yes |
| 90 | RIMKLB |  |  | 726 | 0.645 | 0.4403 | Yes |
| 91 | DHX36 |  |  | 733 | 0.641 | 0.4423 | Yes |
| 92 | NHSL1 |  |  | 734 | 0.641 | 0.4455 | Yes |
| 93 | DUSP16 |  |  | 766 | 0.622 | 0.4424 | No |
| 94 | CTNND2 |  |  | 775 | 0.618 | 0.4439 | No |
| 95 | FIGN |  |  | 853 | 0.578 | 0.4315 | No |
| 96 | PTPRA |  |  | 862 | 0.575 | 0.4327 | No |
| 97 | SPRY2 |  |  | 871 | 0.573 | 0.4340 | No |
| 98 | PAG1 |  |  | 882 | 0.568 | 0.4348 | No |
| 99 | FAM84B |  |  | 892 | 0.564 | 0.4358 | No |
| 100 | ZNF626 |  |  | 918 | 0.553 | 0.4336 | No |
| 101 | NES |  |  | 923 | 0.552 | 0.4356 | No |
| 102 | PHLPP1 |  |  | 977 | 0.533 | 0.4277 | No |
| 103 | ZBTB10 |  |  | 1027 | 0.513 | 0.4205 | No |
| 104 | KIAA1958 |  |  | 1029 | 0.513 | 0.4229 | No |
| 105 | NPAS3 |  |  | 1068 | 0.502 | 0.4178 | No |
| 106 | QKI |  |  | 1078 | 0.500 | 0.4185 | No |
| 107 | ZNF462 |  |  | 1111 | 0.488 | 0.4146 | No |
| 108 | OLIG2 |  |  | 1129 | 0.486 | 0.4136 | No |
| 109 | KIF1B |  |  | 1143 | 0.482 | 0.4134 | No |
| 110 | TANC2 |  |  | 1163 | 0.477 | 0.4120 | No |
| 111 | TBL1XR1 |  |  | 1178 | 0.472 | 0.4116 | No |
| 112 | AUTS2 |  |  | 1211 | 0.462 | 0.4075 | No |
| 113 | KAT6A |  |  | 1212 | 0.462 | 0.4098 | No |
| 114 | DHX40 |  |  | 1228 | 0.457 | 0.4091 | No |
| 115 | RFX3 |  |  | 1241 | 0.453 | 0.4090 | No |
| 116 | EDC3 |  |  | 1245 | 0.453 | 0.4106 | No |
| 117 | PTPRS |  |  | 1253 | 0.451 | 0.4115 | No |
| 118 | VANGL2 |  |  | 1262 | 0.450 | 0.4121 | No |
| 119 | NAALADL2 |  |  | 1268 | 0.448 | 0.4133 | No |
| 120 | SUGP2 |  |  | 1335 | 0.432 | 0.4024 | No |
| 121 | BMP7 |  |  | 1368 | 0.423 | 0.3981 | No |
| 122 | SFXN1 |  |  | 1374 | 0.422 | 0.3992 | No |
| 123 | SOX5 |  |  | 1422 | 0.412 | 0.3919 | No |
| 124 | RNFT2 |  |  | 1426 | 0.410 | 0.3934 | No |
| 125 | FAM110B |  |  | 1440 | 0.405 | 0.3928 | No |
| 126 | IL17RD |  |  | 1446 | 0.403 | 0.3938 | No |
| 127 | DLL1 |  |  | 1463 | 0.399 | 0.3926 | No |
| 128 | FYN |  |  | 1483 | 0.394 | 0.3908 | No |
| 129 | ZNF814 |  |  | 1489 | 0.391 | 0.3917 | No |
| 130 | ELP3 |  |  | 1528 | 0.382 | 0.3861 | No |
| 131 | CREB5 |  |  | 1546 | 0.379 | 0.3846 | No |
| 132 | EDNRB |  |  | 1549 | 0.378 | 0.3861 | No |
| 133 | SP3 |  |  | 1558 | 0.375 | 0.3864 | No |
| 134 | SMARCA4 |  |  | 1584 | 0.369 | 0.3832 | No |
| 135 | PPP1R1C |  |  | 1622 | 0.363 | 0.3777 | No |
| 136 | PTK2 |  |  | 1631 | 0.362 | 0.3779 | No |
| 137 | SEMA6A |  |  | 1633 | 0.362 | 0.3795 | No |
| 138 | GSTA4 |  |  | 1702 | 0.349 | 0.3677 | No |
| 139 | TRAF4 |  |  | 1703 | 0.349 | 0.3695 | No |
| 140 | RFX4 |  |  | 1707 | 0.348 | 0.3706 | No |
| 141 | MARCKS |  |  | 1713 | 0.348 | 0.3713 | No |
| 142 | SP4 |  |  | 1752 | 0.339 | 0.3655 | No |
| 143 | C11orf49 |  |  | 1768 | 0.336 | 0.3641 | No |
| 144 | MKLN1 |  |  | 1769 | 0.336 | 0.3658 | No |
| 145 | GON4L |  |  | 1778 | 0.335 | 0.3659 | No |
| 146 | SPEN |  |  | 1793 | 0.332 | 0.3648 | No |
| 147 | CNOT4 |  |  | 1804 | 0.330 | 0.3644 | No |
| 148 | CHIC2 |  |  | 1819 | 0.328 | 0.3633 | No |
| 149 | SPAG9 |  |  | 1836 | 0.326 | 0.3617 | No |
| 150 | EXOC4 |  |  | 1852 | 0.323 | 0.3603 | No |
| 151 | DAB2IP |  |  | 1867 | 0.319 | 0.3591 | No |
| 152 | PLCE1 |  |  | 1895 | 0.315 | 0.3553 | No |
| 153 | APBA2 |  |  | 1925 | 0.310 | 0.3511 | No |
| 154 | ETV4 |  |  | 1937 | 0.307 | 0.3505 | No |
| 155 | NXPH1 |  |  | 1950 | 0.306 | 0.3496 | No |
| 156 | HMGCS1 |  |  | 1974 | 0.302 | 0.3465 | No |
| 157 | PBX3 |  |  | 1995 | 0.297 | 0.3440 | No |
| 158 | DNAJC18 |  |  | 2006 | 0.295 | 0.3435 | No |
| 159 | CASK |  |  | 2014 | 0.294 | 0.3436 | No |
| 160 | COMMD2 |  |  | 2018 | 0.293 | 0.3444 | No |
| 161 | ARHGEF26 |  |  | 2029 | 0.292 | 0.3439 | No |
| 162 | ZNF507 |  |  | 2041 | 0.290 | 0.3432 | No |
| 163 | TRIM24 |  |  | 2062 | 0.288 | 0.3406 | No |
| 164 | SEZ6L |  |  | 2065 | 0.287 | 0.3416 | No |
| 165 | NDE1 |  |  | 2073 | 0.287 | 0.3417 | No |
| 166 | STRADA |  |  | 2098 | 0.284 | 0.3383 | No |
| 167 | SH3BP4 |  |  | 2193 | 0.269 | 0.3210 | No |
| 168 | UBXN2B |  |  | 2210 | 0.267 | 0.3191 | No |
| 169 | XYLT1 |  |  | 2244 | 0.263 | 0.3139 | No |
| 170 | CNOT2 |  |  | 2256 | 0.261 | 0.3130 | No |
| 171 | ZNF680 |  |  | 2264 | 0.260 | 0.3129 | No |
| 172 | ZSCAN16 |  |  | 2299 | 0.255 | 0.3074 | No |
| 173 | MSL2 |  |  | 2316 | 0.252 | 0.3055 | No |
| 174 | PRKD1 |  |  | 2319 | 0.251 | 0.3063 | No |
| 175 | IGSF11 |  |  | 2330 | 0.250 | 0.3056 | No |
| 176 | HIST1H3G |  |  | 2467 | -0.266 | 0.2799 | No |
| 177 | POLR3B |  |  | 2550 | -0.274 | 0.2650 | No |
| 178 | PRPF19 |  |  | 2604 | -0.283 | 0.2559 | No |
| 179 | WRAP53 |  |  | 2646 | -0.289 | 0.2492 | No |
| 180 | SPATA5 |  |  | 2705 | -0.295 | 0.2391 | No |
| 181 | STX8 |  |  | 2711 | -0.295 | 0.2396 | No |
| 182 | CUEDC1 |  |  | 2825 | -0.309 | 0.2187 | No |
| 183 | PPAT |  |  | 2915 | -0.320 | 0.2026 | No |
| 184 | EZH2 |  |  | 2946 | -0.324 | 0.1982 | No |
| 185 | STX5 |  |  | 2958 | -0.327 | 0.1977 | No |
| 186 | NVL |  |  | 2992 | -0.331 | 0.1928 | No |
| 187 | OSGIN2 |  |  | 3007 | -0.333 | 0.1916 | No |
| 188 | HIST1H2AG |  |  | 3025 | -0.336 | 0.1899 | No |
| 189 | CTNNBL1 |  |  | 3061 | -0.340 | 0.1847 | No |
| 190 | SASS6 |  |  | 3091 | -0.344 | 0.1806 | No |
| 191 | TJP1 |  |  | 3113 | -0.348 | 0.1782 | No |
| 192 | ACD |  |  | 3124 | -0.350 | 0.1779 | No |
| 193 | NUP205 |  |  | 3125 | -0.350 | 0.1797 | No |
| 194 | RPA1 |  |  | 3127 | -0.350 | 0.1812 | No |
| 195 | PNPT1 |  |  | 3254 | -0.369 | 0.1580 | No |
| 196 | EXOSC2 |  |  | 3268 | -0.370 | 0.1573 | No |
| 197 | VAV3 |  |  | 3289 | -0.374 | 0.1552 | No |
| 198 | TMEM209 |  |  | 3326 | -0.379 | 0.1499 | No |
| 199 | TCHP |  |  | 3409 | -0.392 | 0.1356 | No |
| 200 | RAD51C |  |  | 3415 | -0.393 | 0.1365 | No |
| 201 | PRMT5 |  |  | 3442 | -0.397 | 0.1333 | No |
| 202 | AIMP2 |  |  | 3474 | -0.404 | 0.1292 | No |
| 203 | CCDC80 |  |  | 3492 | -0.408 | 0.1278 | No |
| 204 | NDUFA6 |  |  | 3511 | -0.412 | 0.1263 | No |
| 205 | RBM8A |  |  | 3545 | -0.418 | 0.1218 | No |
| 206 | HIST1H2AL |  |  | 3567 | -0.422 | 0.1197 | No |
| 207 | PAICS |  |  | 3594 | -0.427 | 0.1167 | No |
| 208 | RMI1 |  |  | 3668 | -0.438 | 0.1044 | No |
| 209 | TMEM106C |  |  | 3669 | -0.438 | 0.1065 | No |
| 210 | RAD23B |  |  | 3688 | -0.442 | 0.1052 | No |
| 211 | NEDD1 |  |  | 3771 | -0.459 | 0.0912 | No |
| 212 | PEF1 |  |  | 3940 | -0.496 | 0.0603 | No |
| 213 | LFNG |  |  | 3991 | -0.509 | 0.0529 | No |
| 214 | HIST1H3D |  |  | 3996 | -0.510 | 0.0546 | No |
| 215 | HIST1H1E |  |  | 4011 | -0.515 | 0.0544 | No |
| 216 | ITGB3BP |  |  | 4071 | -0.531 | 0.0453 | No |
| 217 | CEP128 |  |  | 4085 | -0.536 | 0.0454 | No |
| 218 | RPF1 |  |  | 4091 | -0.538 | 0.0470 | No |
| 219 | SRP72 |  |  | 4125 | -0.546 | 0.0432 | No |
| 220 | TAF12 |  |  | 4132 | -0.548 | 0.0447 | No |
| 221 | JAG1 |  |  | 4168 | -0.559 | 0.0406 | No |
| 222 | BUB1 |  |  | 4246 | -0.578 | 0.0281 | No |
| 223 | MTHFD1 |  |  | 4274 | -0.590 | 0.0257 | No |
| 224 | RIOK2 |  |  | 4323 | -0.603 | 0.0192 | No |
| 225 | KPNA2 |  |  | 4361 | -0.621 | 0.0149 | No |
| 226 | CEP78 |  |  | 4420 | -0.642 | 0.0066 | No |
| 227 | KIF14 |  |  | 4424 | -0.643 | 0.0092 | No |
| 228 | MRPL52 |  |  | 4429 | -0.644 | 0.0116 | No |
| 229 | NCAPG |  |  | 4568 | -0.716 | -0.0123 | No |
| 230 | AJUBA |  |  | 4605 | -0.735 | -0.0158 | No |
| 231 | HIST1H2AH |  |  | 4645 | -0.762 | -0.0197 | No |
| 232 | USP1 |  |  | 4649 | -0.763 | -0.0166 | No |
| 233 | FANCI |  |  | 4665 | -0.772 | -0.0157 | No |
| 234 | CENPA |  |  | 4683 | -0.788 | -0.0152 | No |
| 235 | NR3C1 |  |  | 4699 | -0.796 | -0.0142 | No |
| 236 | CDC45 |  |  | 4702 | -0.798 | -0.0106 | No |
| 237 | TMPO |  |  | 4790 | -0.851 | -0.0237 | No |
| 238 | PSAT1 |  |  | 4867 | -0.932 | -0.0341 | No |
| 239 | PRR11 |  |  | 4868 | -0.934 | -0.0295 | No |
| 240 | HIST1H1C |  |  | 4877 | -0.940 | -0.0264 | No |
| 241 | FBXO5 |  |  | 4910 | -0.978 | -0.0279 | No |
| 242 | BRCA1 |  |  | 4938 | -1.002 | -0.0283 | No |
| 243 | TMEM100 |  |  | 4976 | -1.053 | -0.0304 | No |
| 244 | UBE2T |  |  | 5000 | -1.091 | -0.0296 | No |
| 245 | MAD2L1 |  |  | 5007 | -1.105 | -0.0253 | No |
| 246 | AURKB |  |  | 5016 | -1.118 | -0.0213 | No |
| 247 | ADAMTS6 |  |  | 5017 | -1.121 | -0.0158 | No |
| 248 | TPX2 |  |  | 5049 | -1.184 | -0.0160 | No |
| 249 | CCDC34 |  |  | 5111 | -1.316 | -0.0216 | No |
| 250 | CDK1 |  |  | 5164 | -1.512 | -0.0244 | No |
| 251 | TLE3 |  |  | 5206 | -1.684 | -0.0242 | No |
| 252 | HIST1H1B |  |  | 5223 | -1.811 | -0.0184 | No |
| 253 | HIST1H1D |  |  | 5231 | -1.871 | -0.0105 | No |
| 254 | HIST1H4C |  |  | 5249 | -2.081 | -0.0036 | No |
| 255 | PTCH1 |  |  | 5261 | -2.318 | 0.0058 | No |
Table: GSEA details [plain text format]

  

Fig 2: SINGH\_SOX2\_UPREGULATED\_TRANSCRIPTS\_PMID\_28122245: Random ES distribution      
 Gene set null distribution of ES for **SINGH\_SOX2\_UPREGULATED\_TRANSCRIPTS\_PMID\_28122245**
